# Enhanced human sensorimotor integration via self-modulation of the somatosensory activity

**DOI:** 10.1016/j.isci.2025.112145

**Published:** 2025-03-03

**Authors:** Seitaro Iwama, Takamasa Ueno, Tatsuro Fujimaki, Junichi Ushiba

**Affiliations:** 1 Department of Biosciences and Informatics, Faculty of Science and Technology, Keio University, Yokohama, Kanagawa, Japan; 2 Graduate School of Science and Technology, Keio University, Yokohama, Kanagawa, Japan

**Keywords:** Neuroscience, Behavioral neuroscience, Cognitive neuroscience

## Abstract

Motor performance improvement through self-modulation of brain activity has been demonstrated through neurofeedback. However, the sensorimotor plasticity induced through the training remains unclear. Here, we combined individually tailored closed-loop neurofeedback, neurophysiology, and behavioral assessment to characterize how the training can modulate the somatosensory system and improve performance. The real-time neurofeedback of human electroencephalogram (EEG) signals enhanced participants’ self-modulation ability of intrinsic neural oscillations in the primary somatosensory cortex (S1) within 30 min. Further, the short-term reorganization in S1 was corroborated by the post-training changes in somatosensory evoked potential (SEP) amplitude of the early component from S1. Meanwhile those derived from peripheral and spinal sensory fibers were maintained (N9 and N13 components), suggesting that the training manipulated S1 activities. Behavioral evaluation demonstrated improved performance during keyboard touch-typing indexed by resolved speed-accuracy trade-off. Collectively, our results provide evidence that neurofeedback training induces functional reorganization of S1 and sensorimotor function.

## Highlights

• EEG neurofeedback improved self-modulation of SM1 neural oscillations within 30 min
• S1 reorganization was confirmed by increased SEP amplitude
• Improved touch-typing shows neurofeedback’s role in S1 reorganization for skill enhancement

## Graphical abstract

**Figure:**
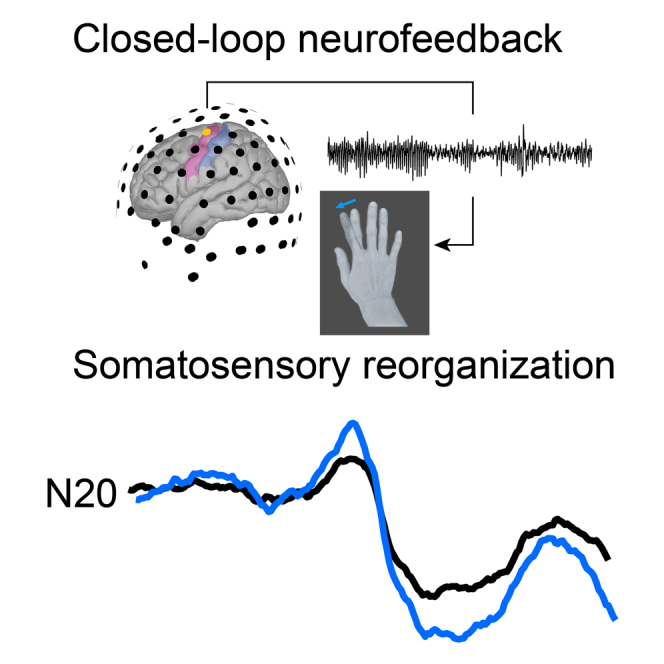

## Introduction

Successful motor control largely relies on somatosensory function to correct ongoing and subsequent behaviors.^1^^,^^2^^,^^3^^,^^4^ For instance, human beings perform dexterous movements with remarkable accuracy during cultural and occupational situations, highlighting the critical importance of somatosensory function for motor control.^5^^,^^6^ The neural mechanisms underlying sensorimotor integration skills have been explored using experimental tasks such as sequential finger tapping,^7^ pinching,^8^^,^^9^ and grasping.^10^ Moreover, specialized somatosensory function is a hallmark of fields requiring motor skills, such as musical and athletic performance, suggesting that intense, continuous, and demanding training reorganizes the neural circuits behind accurate sensory processing.^5^^,^^6^ These studies demonstrate that the distributed sensorimotor network is tuned and engaged in processing information, constantly interacting with descending motor output and ascending sensory input.

By engaging the neural repertoires involved in a motor task without actually executing it, improvements in motor performance have been demonstrated through self-modulation of neural activities using neurofeedback experiments. During the training, combined with motor imagery, neuroimaging data including invasive electrophysiological recordings indicate activation of the primary somatosensory and motor cortices (S1 and M1, respectively), which are putatively implicated during movement initiation and preparation.^11^^,^^12^^,^^13^^,^^14^^,^^15^ However, whether the neurofeedback intervention induces the sensory plasticity and how it contributes to the sensorimotor performance improvement remain unclear.

**Figure 1:**
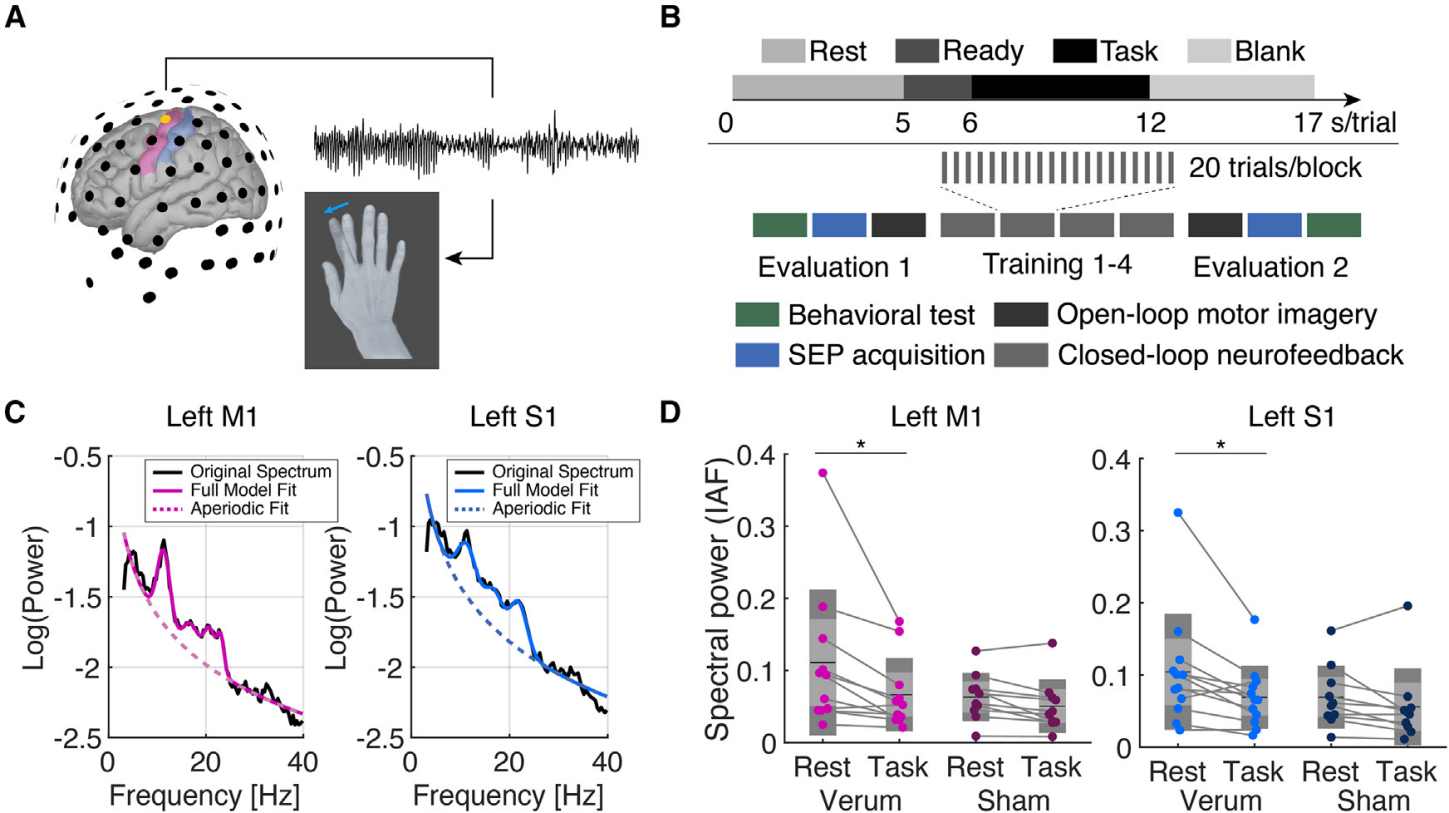
Closed-loop sensorimotor activity feedback experiment (A) Schematic of the experiment. Real-time processed scalp electroencephalogram (EEG) signals derived from the left sensorimotor cortex were associated with the movement of right index finger abduction shown on the screen. The range of motion represents EEG spectral power attenuation during motor imagery task. (B) Experiment protocol. The upper panel indicates time course of a motor imagery trial, which comprised of four periods: rest, ready, task, and blank. The entire trial lasted 17 s, and 20 trials were conducted in a block. The experimental procedure includes two evaluation (open-loop) and four training (closed-loop) blocks. These blocks were flanked with behavioral test and somatosensory evoked potential acquisition. (C) Power spectral density (PSD) plots for the left primary motor and sensory cortices (M1 and S1). The black solid lines indicate the original spectrum, and the colored indicate the full model fit acquired by the specparam algorithm.^17^ The dotted lines represent the aperiodic fit. (D) Spectral power peak at individual alpha frequency (IAF) for the left M1 and S1. We found a significant main effect of condition in both left M1 and S1 data (M1: *F*(1,20) = 8.94, *p* = 0.008, *η*^2^ = 0.051; S1: *F*(1,20) = 9.62, *p* = 0.006, *η*^2^ = 0.045), but not the interaction of condition and group effects (M1: *F*(1,20) = 2.77, *p* = 0.11; S1: *F*(1,20) = 1.95, *p* = 0.18) or the main effect of group (M1: *F* (1,20) = 1.59, *p* = 0.22, S1: *F*(1,20) = 1.08, *p* = 0.31). ∗: corrected *p* < 0.05, *N* = 22.

In the present study, we hypothesized that closed-loop neurofeedback training would enhance subsequent motor performance by rehearsing task-related activation of the primary somatosensory and motor cortices and promoting functional remodeling of the somatosensory pathways. To this end, we asked if induced sensorimotor plasticity through the short-term motor imagery training is boosted by the closed-loop feedback and if it manipulates S1 reactivity, as assessed by the somatosensory evoked potential (SEP) and behavioral test for sensorimotor processing in healthy humans. To train for the self-modulation of sensorimotor neural excitability, we employed individually tailored closed-loop motor imagery. During the closed-loop motor imagery exercise, virtual finger movements were provided in real time, based on estimates of the sensorimotor rhythm (SMR) amplitude^16^ (Figure 1A). If the self-modulated SMR can manipulate the somatosensory pathways, the SEP amplitude change would accompany SMR modulation.

## Results

### Training-induced neural activity modulation was frequency and region specific

We recruited 39 participants for the main experiment, and data from 22 participants were subjected to main analysis. We excluded relatively larger sample size due to the high drop rate for the aversion to the median nerve stimulation and compromised quality for the SEP data (see STAR Methods for the criteria).

The participants were asked to perform motor imagery of index finger abduction and instructed to kinesthetically imagine contracting the right first dorsal interosseous muscle during which the joint angle of the index finger displayed on a monitor was controlled based on the spectral power of electroencephalogram (EEG) signals at individual alpha frequency (IAF), derived from the contralateral sensorimotor cortex (i.e., C3; Figure 1A). Namely, the finger was configured to show abduction when the IAF spectral power at the C3 channel was attenuated compared to a 5-s resting state period before a 6-s motor imagery period (Figure 1B).

The participants were allocated to one of two groups: the verum group, where they observed the images of finger movement linked with their own scalp EEG data, and the sham group, where they observed those linked with previous data from others (verum: *N* = 12; sham: *N* = 10). The group allocation was randomized and single blinded throughout the study. No participants reported adverse effects throughout and after the experiment.

As a first step of the offline analysis, we estimated the cortical source using the high-density EEG signals and differently assessed the M1 and S1 activities.^18^^,^^19^ After calculating the power spectral density (PSD) derived from the contralateral M1 and S1, IAF was identified using the spectral parameterization algorithm (Specparam^17^; Figure 1C). The spectral power at the IAF was compared with a mixed repeated-measures ANOVA (rmANOVA) with condition (rest or task) and group (verum or sham) effects (Figure 1D). We found a significant main effect of condition in both left M1 and S1 data (M1: *F*(1,20) = 8.94, *p* = 0.008, *η*^2^ = 0.051; S1: *F*(1,20) = 9.62, *p* = 0.006, *η*^2^ = 0.045), but not the interaction of condition and group effects (M1: *F* = 2.77, *p* = 0.11; S1: *F* = 1.95, *p* = 0.18) or the main effect of group (M1: *F*(1,20) = 1.59, *p* = 0.22, S1: *F*(1,20) = 1.08, *p* = 0.31). *Post hoc* t tests revealed a significant difference between the rest and task periods during training blocks in the two areas in the verum group (paired t test with Bonferroni correction, M1: *t*(10) = 3.37, corrected *p* = 0.019, *d* = 0.72; S1: *t*(10) = 3.34, corrected *p* = 0.02, *d* = 0.62). Since attenuated spectral power around SM1 reflects desynchronized rhythmic activities due to cortical excitability change,^20^^,^^21^ the task-related power attenuation indicates that the self-modulation of SM1 activity was successfully induced during motor imagery training.^22^^,^^23^ The spectral power modulation was found in both groups since motor imagery activates sensorimotor cortex without closed-loop feedback. A significant main effect of group was also found in the beta-band power but not evident in the theta band (Figures S1A and S1B). In addition, the between-group difference in IAF and the broadband aperiodic component were not evident, and the spectral power derived from the S1 and M1 was significantly correlated (Pearson’s correlation test: *r* = 0.698 ± 0.25, *p* < 0.001). These findings suggest that the oscillatory spectral power related to the SMR component used in the real-time feedback, both originated from the sensorimotor cortex, was modulated during the closed-loop training.

To confirm whether the periodic component of the PSD was modulated during the training, the spectral peak height at IAF was compared. A significant group difference during the rest period in training blocks was found (Figure S2A, two-sample t test, M1: *t*(20) = 2.79, *p* = 0.0014, *d* = 1.15; S1: *t*(20) = 2.74, *p* = 0.0015, *d* = 1.14) while no other frequency bands exhibited systematic between-group difference (Figures S2B and S2C). Collectively, the periodic power change indicates that closed-loop SMR control manipulates neural population oscillating at the targeted frequency.

### Modulation depth of the sensorimotor oscillatory activities was augmented by the closed-loop prosthetic control training

**Figure 2:**
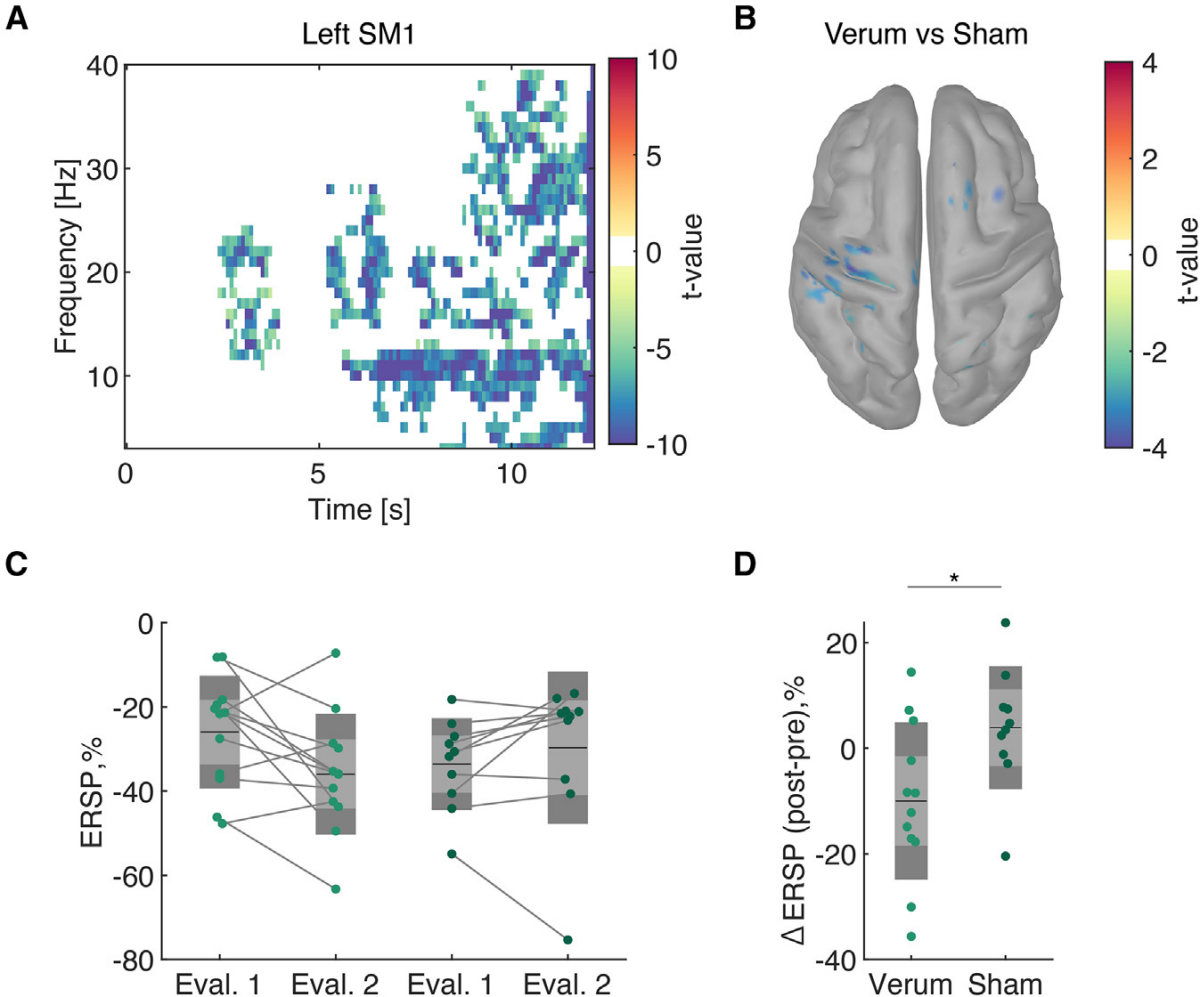
Time-frequency representations and comparison of event-related spectral perturbations between verum and sham groups (A) Time-frequency representation of power changes in the left SM1 during the motor imagery task. The clusters exceeding the significance threshold (*p* < 0.0001) were colored with their t values. Negative t values represent significant spectral power attenuation compared to the rest period. (B) Spatial distributions of significant t values indicating difference between verum and sham groups. (C) Comparison of event-related spectral perturbation (ERSP) during evaluation 1 and evaluation 2 for verum and sham groups. Each dot represents an individual participant, and lines connect the same participant’s data across evaluations. The dark gray areas, light gray areas, and the black line represent 1 SD, 95% confidence interval, and mean values, respectively. (D) Changes in ERSP from evaluation 1 to 2 for the verum and sham groups in the left M1 and S1. A significantly prominent spectral power attenuation was found in the left SM1 in the verum group. The rmANOVA revealed a significant interaction of group and time effects in the SM1 ERSP magnitude in the trained hemisphere (rmANOVA, *F*(1,20) = 5.88, *p* = 0.025, *η*^2^ = 0.06). *Post hoc* two-sample t tests for the changes in ERSP magnitude from evaluation 1 to 2 revealed that the verum group exhibited a significant decrease in ERSP magnitude compared to the sham group (two-sample t test, *t*(20) = −2.43, *p* = 0.025, *d* = 1.04). ∗: corrected *p* < 0.05, *N* = 22.

Given that the verum group indicated successful SMR modulation during training, we evaluated the endogenous modulation depth of SMR during the motor imagery task in the open-loop motor imagery blocks (Figure 2). To this end, we quantified event-related spectral perturbation (ERSP) magnitude at IAF for each participant, which represents the within-trial normalized magnitude of spectral power modulation-calculated rest period data as baseline. A significant decrease in the ERSP magnitude around 8–30 Hz was found in both left SM1, indicating that motor imagery tasks induce significant self-modulated SMR in the verum group (Figure 2A, one-sample t test with Bonferroni correction). The broad band spectral power modulation across 8–30 Hz is consistent with previous reports on event-related desynchronization (ERD) of SMRs as the sensorimotor mu rhythm exhibits harmonic responses.^24^^,^^25^^,^^26^ The significant areas of between-group comparisons for SMR were localized around SM1 in the left hemisphere (Figure 2B, two-sample t test with cluster-level false-discovery correction). Meanwhile, the sham group did not exhibit systematic modulation across participants. Collectively, participants in the verum group performed kinesthetic motor imagery accompanied with the significant activity modulation in SM1, despite its difficulty for the naive participants.^20^^,^^21^^,^^22^

Next, to compare the task-related modulation depth of SMR magnitude, the block-average ERSP magnitudes were subjected to a mixed rmANOVA (Figure 2C). The rmANOVA revealed a significant interaction of group and time effects in the SM1 ERSP magnitude in the trained hemisphere (rmANOVA, *F*(1,20) = 5.88, *p* = 0.025, *η*^2^ = 0.06). *Post hoc* two-sample t tests for the changes in ERSP magnitude from evaluation 1 to 2 revealed that the verum group exhibited a significant decrease in ERSP magnitude compared to the sham group (Figure 2D, two-sample t test, *t*(20) = −2.43, *p* = 0.025, *d* = 1.04). The robustness of the present finding was confirmed by rmANOVA with aligned rank transform^27^ (rmANOVA with aligned rank transform: *F*(1,20) = 6.92, *p* = 0.016, *η*^2^ = 0.09), and that for the intention-to-treat population, whose data during evaluation blocks were available, was conducted (Figure S3). This suggests that short-term kinesthetic motor imagery induced enhanced SM1 activities without any somatosensory feedback, consistent with the recent findings in the non-invasive functional neuroimaging data during motor preparation^14^^,^^15^ as well as the single neuronal activity derived from S1.^28^^,^^29^

### Neurofeedback-induced somatosensory evoked response modulation

**Figure 3:**
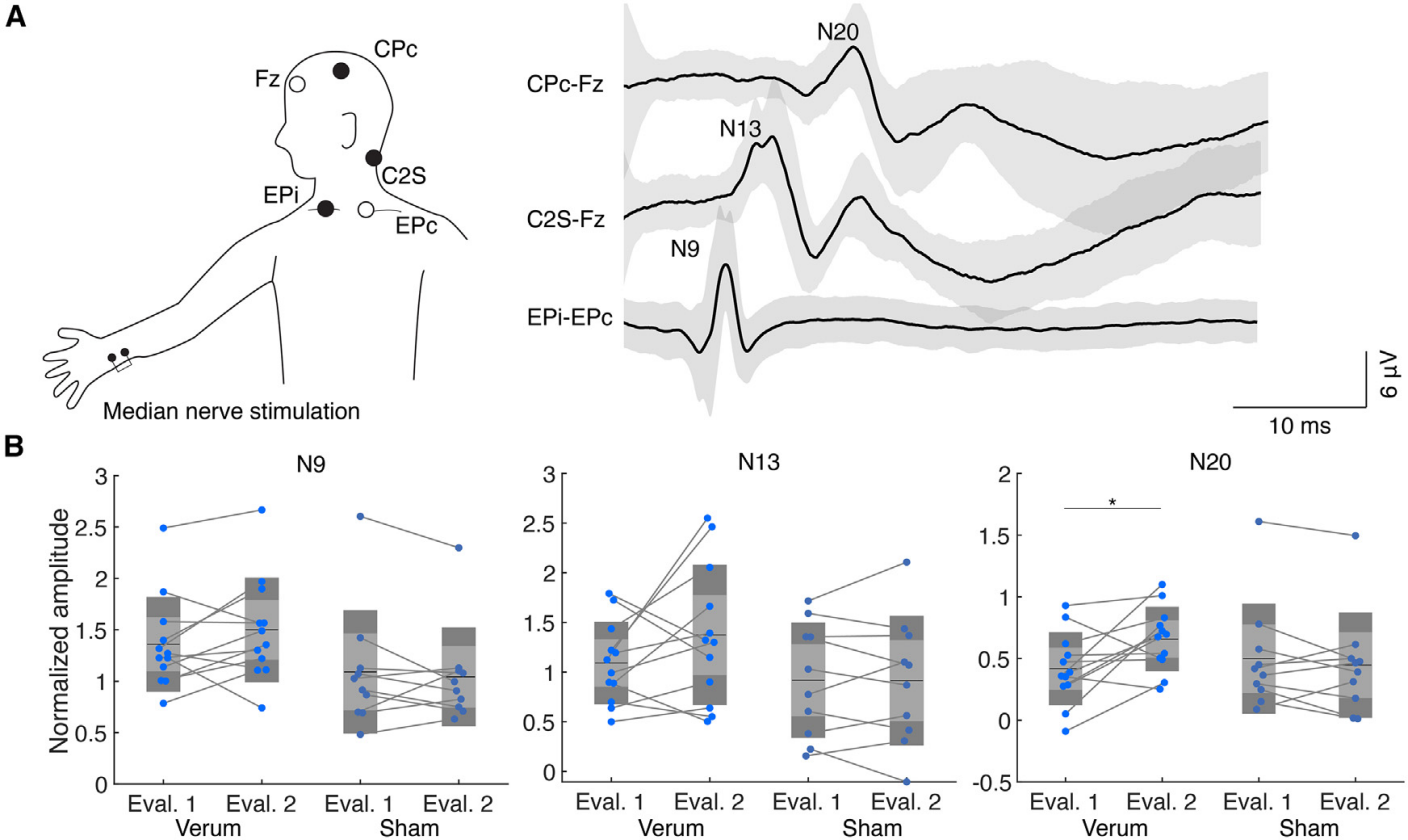
Somatosensory evoked potential results (A) Schematic representation of electrode placement for recording SEPs elicited by right median nerve stimulation. Electrode positions were Fz, CPc, C2S, EPi, and EPc. The SEP waveforms show typical peaks (N20, N13, and N9) recorded at different electrode montages (CPc-Fz, C2S-Fz, and EPi-EPc). The gray shade indicates 1 standard deviation. (B) Normalized amplitudes of SEP components (N9, N13, and N20) during evaluation 1 and evaluation 2 for the verum and sham groups. Each dot represents an individual participant, and lines connect the same participant’s data across evaluations. A significant interaction of time and group effect was found in the N20 component, and *post hoc* t test with Bonferroni correction revealed a significant increase in amplitude in the verum group (rmANOVA: N9: *F*(1,20) = 2.18, *p* = 0.16; N13: *F*(1,20) = 1.75, *p* = 0.20; N20: *F*(1,20) = 6.49, *p* = 0.019, *η*^*2*^ = 0.04; *post hoc* t test for N20 data: paired t test with Bonferroni correction, verum: *t*(11) = −3.10, corrected *p* = 0.033, *d* = −0.68, sham: *t*(9) *=* 0.614, corrected *p* = 1.0). ∗: corrected *p* < 0.05, *N* = 22.

To further investigate the somatosensory reactivity manipulated by the closed-loop motor imagery intervention, we assessed the changes in SEP magnitude derived from multiple locations (Figure 3A). The ascending somatosensory signals evoked by right median nerve stimulation were visualized as N9, N13, and N20 components. Each of the components reflect difference neuronal reactivity^30^^,^^31^: N9 represents the brachial plexus response, N13 represents the cervical spinal cord response, and N20 represents the early component of the S1 response, specifically Brodmann’s 3b area.^31^^,^^32^

To identify changes in SEP amplitude induced by the training, three components were subjected to the rmANOVA (Figure 3B). We found a significant interaction of time and group in the N20 component (rmANOVA, N9: *F*(1,20) = 2.18, *p* = 0.16; N13: *F*(1,20) = 1.75, *p* = 0.20; N20: *F*(1,20) = 6.49, *p* = 0.019, *η*^2^ = 0.04). *Post hoc* t tests revealed a significant increase in the amplitude in the verum group (paired t test with Bonferroni correction, *t*(10) = −3.10, *p* = 0.033, *d* = −0.68). Meanwhile, other components did not exhibit significant interaction or main effects (all statistical results shown in supplemental information). Additionally, we explored the relationship between SEP modulation and SMR-ERD change. We found a significant positive correlation between ERD and SEP modulation (Pearson’s correlation: *r* = −0.461, *p* = 0.031, see Figure S4 for full statistical results).

### Improved sensorimotor task performance through motor imagery practice

The closed-loop motor imagery training without any somatosensory feedback manipulated both oscillatory SM1 activity modulation during motor imagery in open-loop condition and S1 reactivity to the somatosensory stimuli. If the motor imagery training can act as the rehearsal of SM1 activation during motor tasks, the training could augment sensorimotor processing in which both M1 and S1 are actively involved.

To corroborate the hypothesis, we tested the behavioral performance during a motor task requiring sensorimotor integration process. Specifically, we employed a keyboard touch-typing task^33^^,^^34^ to quantify post-training meditation of speed-accuracy trade-off. Since the somatosensory function can be used to execute rapid and accurate finger movement, we calibrated the task difficulty to adjust the individual typing performance. We found no evidence in the significant difference group-level task difficulty, measured by the rank and number of letters of selected words (two-sample t test, word rank: *t*(20) = 1.30, *p* = 0.20, $\log\;BF_{10}=0.43$; word letters: *t*(20) = 1.94, *p* = 0.06, $\log\;BF_{10}=0.32$). Participants underwent 5 blocks of the typing task, with 30 words presented in each block, using a keyboard without key printing.

**Figure 4:**
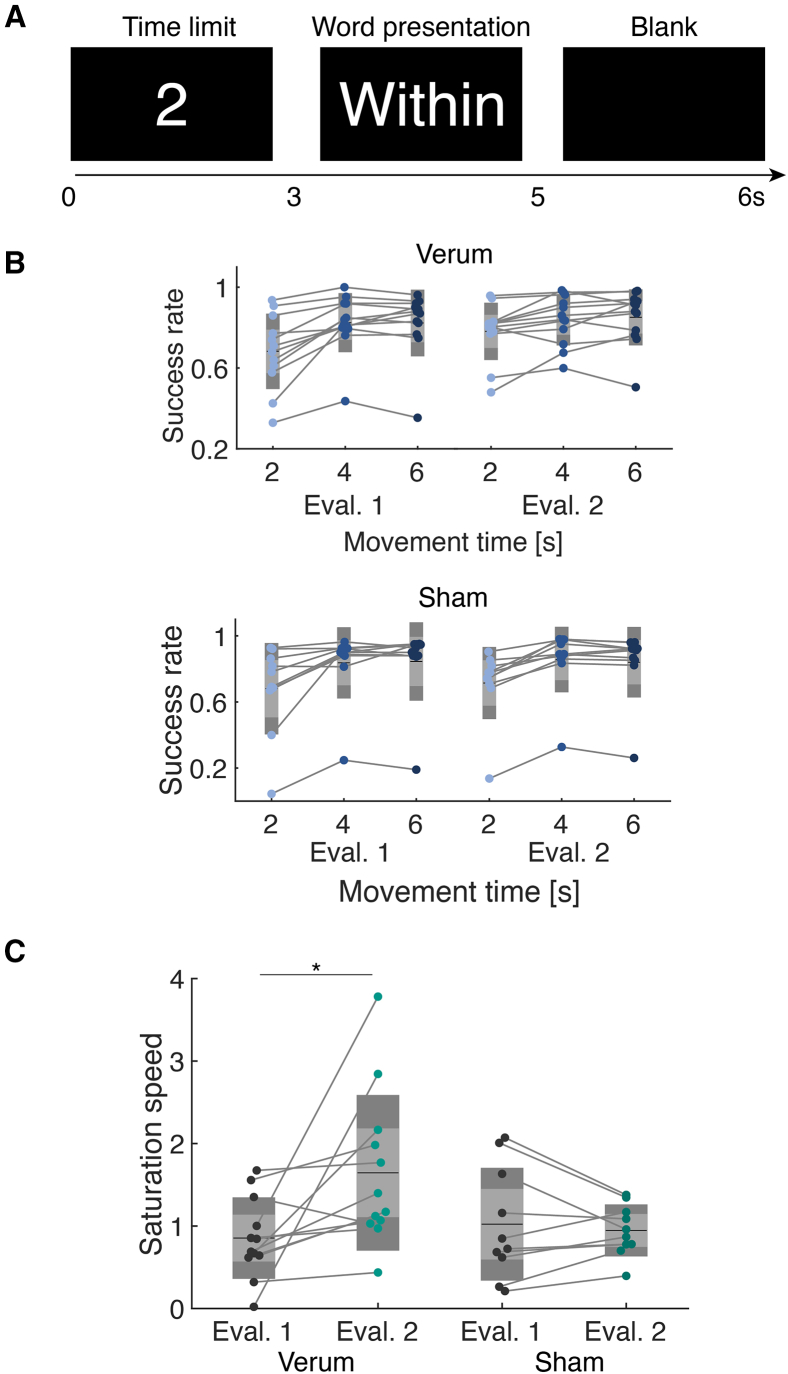
Behavioral test results (A) Schematic representation of a single trial in the experiment. The trial includes three periods: a 2-s time limit display, word presentation, and a 1-s blank. The duration of word presentation finished when participants failed to type the correct key. (B) Success rates of participants in the verum and sham groups during evaluation 1 and evaluation 2 at different movement times (2, 4, and 6 s). Each dot represents an individual participant, and lines connect the same participant’s data across evaluations. (C) Comparison of saturation speed (the speed at which the success rate plateaus) between evaluation 1 and evaluation 2 for the verum and sham groups. A mixed rmANOVA revealed significant interaction of time and group effects. The *post hoc* t test with Bonferroni correction revealed a significant increase in the verum group (rmANOVA: *F*(1,20) = 6.06, *p* = 0.023, *η*^2^ = 0.096; t test: *t*(11) = −3.31, *p* = 0.02, *d* = −1.21). ∗: corrected *p* < 0.05, *N* = 22.

A trial began with the time limit presentation; the three conditions (2, 4, and 6 s) were presented in pseudo-randomized order (Figure 4A). The word presentation period lasted for the time limit, and the blank period followed. A trial was counted as success when participants typed the given word without an error in each time limit. We calculated the accuracy for each condition in evaluation 1 and 2 (Figure 4B). Then, the speed-accuracy trade-off curve was estimated by fitting the following formula, y=aexp(−bx)+c where y and x represent the accuracy and condition, respectively. We fitted parameters a,b,andc, and the saturation speed b was subjected to a mixed rmANOVA with time and group effects (Figure 4C). A significant interaction of time and group effects was found (*F*(1,20) = 6.06, *p* = 0.023, *η*^2^ = 0.096). The *post hoc* t test revealed a significant increase in the saturation speed in the verum group after training (paired t test with Bonferroni correction, *t*(11) = −3.31, corrected *p* = 0.02, *d* = −1.21), suggesting that the sensorimotor processing was modulated through the closed-loop motor imagery training. Meanwhile, we did not find a systematic change in other fitted parameters (Figure S5).

## Discussion

In the present study, we sought the neurofeedback effects on the somatosensory system and its association with the motor performance improvement. The group allocated to the veritable neurofeedback showed enhanced voluntary controllability of neural signals derived from the SM1, somatosensory reactivity, and speed-accuracy balance during touch-typing behavior. This study provides the first demonstration that somatosensory reorganization can be induced by endogenous neural activity through neurofeedback, extending previous findings that externally induced somatosensory plasticity can be enhanced by manipulating neural oscillations.^35^ Our finding that touch-typing performance can be improved through self-modulation of sensorimotor activity holds potential significance for fields such as sports, music, and occupational training, where sensorimotor integration is a critical skill.

Human beings are adept at imagining the consequences of their actions and refining their motor plans to achieve manual dexterity. This skill perhaps leads to a rich repertoire of motor patterns and, ultimately, to human prosperity. To this end, humans employ kinesthetic motor imagery to engage the neural repertoires involved in a motor task without execution. Regardless of the absence of somatosensation, neuroimaging data including invasive electrophysiological recordings indicate task-related activation of the SM1 during motor imagery, putatively implicated in simulating the representation during movement initiation and preparation.^12^^,^^13^^,^^14^^,^^36^ However, the direct link between the somatosensory activation during the kinesthetic motor imagery and subsequent performance improvement was unclear.

In the present study, we demonstrated that closed-loop neurofeedback training specifically modulated the targeted sensorimotor cortex and significantly improved behavioral performance during a touch-typing task as indicated by the speed-accuracy trade-off. Furthermore, the skill acquisition of sensorimotor activity self-modulation resulted in a significant change in the N20 component of SEPs, indicating enhanced sensory reactivity in the S1.^6^^,^^37^^,^^38^ These findings suggest enhanced somatosensory function, on which successful motor control is highly dependent.^6^ We also found that the training effects in part generalized to the open-loop blocks during evaluation, where no feedback was provided. It indicates that the self-modulation ability of population-level S1 activity trained through the closed-loop motor imagery was not confined to the feedback blocks alone but also extended to the open-loop condition, and the training maintained significant SMR modulation depth. This suggests that the training induced lasting changes in sensorimotor cortex excitability that persisted beyond the immediate training context.^39^^,^^40^ These observations are consistent with reports that manipulating M1 activity using a closed-loop neurofeedback paradigm influences corticospinal excitability,^16^^,^^39^^,^^41^^,^^42^ indicating the potential utility of closed-loop motor imagery to enhance sensorimotor activities before the actual sensorimotor behavior is required.^43^

Our finding that touch-typing task performance can be improved via the self-modulation of sensorimotor activities is of potential importance for the fields of sports, musical, and occupational scenes as sensorimotor integration ability quantified by the present task is ubiquitously required in these areas. Although the success rate did not show changes with training, it may not fully represent underlying improvements due to its susceptibility to strategy adjustments rather than pure skill enhancement. Moreover, a direct comparison of success rates does not account for the multivariate relationships between success rates across time limits. Meanwhile, saturation speed to evaluate performance improvement was less influenced by compensatory strategies.

We found that few participants in the sham group exhibited self-regulation of SMR-ERD during the post-evaluation block. We posit that learning of SM1 activity regulation is possible for those in the sham group because of the neurofeedback training, in which we leveraged kinesthetic motor imagery as an explicit strategy, and motor imagery activates sensorimotor cortex without closed-loop feedback.^20^^,^^21^ Thus, the neurofeedback system could have played an adjuvant role for the self-modulation of SMR-ERD. Moreover, the yoked-sham neurofeedback design does not necessarily have a detrimental effect as we used the scalp EEG signals during motor imagery from others, which in general exhibited SMR-ERD. Hence, the participants allocated to the sham group exhibit learning of spectral power modulation of scalp EEG during motor imagery. Nevertheless, we posit that the effect of neurofeedback training on the somatosensory representation and motor performance was driven by the learning observed in the evaluation blocks (i.e., motor imagery conditions), which was specific to the verum group (Figure 2D). The veritable closed-loop neurofeedback training can augment the self-modulation of sensorimotor activities even in the absence of the feedback.

### Limitations of the study

Our study has three limitations: (1) the single-blind study design, (2) the absence of an open-loop (i.e., no feedback) group or a group receiving feedback from different regions, and (3) the limited duration of the training period. First, the single-blind design may introduce bias, as experimenters were aware of their group assignments. While this design might influence subjective judgments, it is unlikely to affect the objective neurophysiological measures such as SEPs and SMR, which were the primary outcomes of this study. Additionally, the randomization of participants into groups helps mitigate potential biases, and they were not informed about the existence of the other group. Therefore, it is not likely that the placebo and nocebo effects lead to the result. Second, the absence of a purely open-loop group means it is unable to entirely separate the effects of motor imagery from those of feedback. Despite this, the significant differences observed between the verum and sham groups provide strong evidence that the feedback component plays a crucial role in modulating SMR-ERD. Relatedly, the absence of the group that receives neurofeedback of control sites is necessary to conclude that the volitional regulation of SM1 activity has a unique effect for reorganization of the somatosensory system.^44^ However, our study can contribute to the extension of the present understanding about the SMR-ERD neurofeedback employed in a variety of situations including rehabilitation, sports, and brain-computer interface operation. Future studies incorporating a no-feedback group and alternative feedback group would clarify the specific contributions of feedback. Last, the short duration of the training period limits our understanding of long-term effects and consolidation of sensorimotor skills. However, the robust changes observed within this brief period suggest that even short-term interventions can induce apparent neural plasticity. Extending the duration of the training in future research will help determine the sustainability of these effects and their potential for long-term rehabilitation outcomes.

## Resource availability

### Lead contact

Further information and requests for resources should be directed to and will be fulfilled by the lead contact, Seitaro Iwama (iwama@bio.keio.ac.jp).

### Materials availability

This study did not generate new unique reagents.

### Data and code availability

• All data used in the present study are shown in the figures and supplemental information.
• Code related to the present study is deposited to the GitHub repository https://github.com/Junichi-Ushiba-Laboratory/enhanced-human-sensorimotor-integration-via-self-modulation-of-the-somatosensory-activity.
• Any additional information required to reanalyze the data reported in this paper is available from the lead contact upon request.

## Acknowledgments

We thank Dr. F. Iwane for feedback on this paper. This study was supported by 10.13039/501100002241JST, PRESTO grant number JPMJPR23I1, Japan, and 10.13039/501100001691JSPS KAKENHI grant number 20H05923, Japan. The authors thank Shoko Tonomoto, Aya Kamiya, and Sayoko Ishii for their assistance.

## Author contributions

Conceptualization, S.I. and J.U.; methodology, S.I. and T.U.; software, S.I., T.U., and T.F.; validation, S.I.; formal analysis, S.I.; investigation, S.I., T.U., and J.U.; resources, S.I. and T.F.; data curation, S.I., T.U., and J.U.; writing – original draft, S.I.; writing – review and editing, T.F., T.U., and J.U.; visualization, S.I.; supervision, S.I. and J.U.; project administration, S.I. and J.U.; funding acquisition, S.I. and J.U.

## Declaration of interests

J.U. is the founder and representative director of the university startup company LIFESCAPES Inc., involved in the research, development, and sales of rehabilitation devices, including the brain-computer interfaces. He receives a salary and holds shares in LIFESCAPES Inc. S.I. and T.F. receive a salary from the company. The company did not have any relationship with the device or setup used in this study.

## STAR★Methods

### Key resources table

**Table:** 

REAGENT or RESOURCE	SOURCE	IDENTIFIER
**Experimental models: Organisms/strains**		

Healthy human volunteers	This manuscript	N/A

**Software and algorithms**		

Specparam	Donoghue T, Haller M, Peterson EJ, Varma P, Sebastian P, Gao R, Noto T, Lara AH, Wallis JD, Knight RT, Shestyuk A, & Voytek B	https://github.com/fooof-tools/fooof
Brainstorm	Tadel F, Baillet S, Mosher JC, Pantazis D, Leahy RM.	https://github.com/brainstorm-tools/brainstorm3

### Experimental model and study participant details

We recruited 39 participants for the main experiment and data from 22 participants were subjected to main analysis (17 males and 5 females; age: 23.62 ± 3.4, all right-handed, ethnically Japanese), and randomly allocated to the verum or sham group. No sex-dependent effects were tested in the present study. We excluded relatively larger sample size due to the high drop rate for the aversion to the median nerve stimulation before completing Evaluation 1. Written informed consent to participate in the present study was obtained from every participant. All experiment protocol was approved by the Ethics Committee of the Faculty of Science and Technology, Keio University (IRB approval number: 2023-128).

### Method details

#### Experiment procedure

The experiment was performed in accordance with the Declaration of Helsinki. No adverse effects were reported throughout study. The experiment comprised three phases: pre-training evaluation (Evaluation 1), training and post-training evaluation (Evaluation 2) as shown in Figure 1B. In the evaluation phase, participants underwent behavioral test, SEP acquisition, and the open-loop motor imagery block. In the training phase, participants experienced four blocks of closed-loop motor imagery training.

#### Behavioral test

As a keyboard typing task, we used the English words listed in the General Service List^45^ (GSL), ensuring that non-native English speakers could recognize the words within the movement time limit. Participants performed a typing speed assessment before Evaluation 1 using a web service to quantify their characters per minute (CPM, https://typing-speed-test.aoeu.eu/). The CPM was used to determine the maximum word length. We used words with 80-100% of the characters that each participant was capable of typing within the given movement time for each condition.

#### Somatosensory evoked potential acquisition

The SEP data were acquired to evaluate the reactivity of S1 by stimulating the right median nerve using Neuropack X1 (Nihon Kohden, Tokyo, Japan). The stimulation trigger and analog data derived from electrodes on CPc, Fz, C2S, EPi and EPc were sampled using the setup and recorded using an analog-digital converter (USB-6259, National Instruments, Austin, U.S.A.) at the sampling rate of 10000 Hz. The acquired signal data were filtered using a highpass filter (3 Hz) and a lowpass filter (500 Hz).^30^

The stimulation intensity was adjusted to the individual motor threshold (MT). To determine MT, the sensory threshold was firstly identified by increasing the intensity until participants were aware of stimulus presentation, Then, MT was identified at the minimum intensity which elicit stimulation-evoked thumb movement. The MT determined in the Evaluation 1 was also used in Evaluation 2 to avoid confounding the sensory plasticity with stimulation intensity changes. In each Evaluation block, 1000 stimulations were presented at 2 Hz frequency.

#### Kinesthetic motor imagery task

In the motor imagery blocks, we asked participants to perform kinesthetic motor imagery, that is to mentally rehearse body movement.^46^ The participants performed the imagery of right index finger abduction. At the beginning of the experiment, participants were verbally instructed how to perform the kinesthetic motor imagery. The content of verbal instruction was consistent across participants using a script (See *Script for the instruction of kinesthetic motor imagery* in supplemental information).

In the open- and closed-loop motor imagery blocks, participants performed 20 trials in which a 5-second rest and a 1-second ready period were followed by a 6-second task period. During the task periods, participants were asked to perform the kinesthetic motor imagery as instructed. The absence of overt movement during the training was confirmed by the visual confirmation.

#### Real-time sensorimotor rhythm amplitude neurofeedback

In the closed-loop blocks, participants received the neurofeedback of SMR amplitude in real-time through virtual right index finger movement shown on the display in front of participants.^22^^,^^40^^,^^43^ The spectral power of SMR was real-time calculated and associated with the Metacarpo Phalangeal joint angle (Figure 1A). At the beginning of the experiment, we captured the abduction of the right index finger using a video camera and extracted 20 frames from the movie data. The frame of the more abducted finger was presented if spectral power attenuation of SMR was found in the electrode over the left SM1.

To determine the frame to present, we analyzed the online acquired scalp EEG data. The experiment setup was employed in the previous EEG-based neurofeedback studies.^22^^,^^40^^,^^47^ During the motor imagery blocks, we used the Electrical Geodesics system with 128-channel HydroCel Geodesic Sensor Net (GES 400, Electrical Geodesics, Inc.). EEG electrode impedance levels were kept below 50 kΩ throughout the experiment. A 100 Hz online low pass filter was applied to recorded data at a sampling rate of 1000 Hz.

The EEG signals were subjected to online analysis to calculate SMR magnitude around contralateral SM1. The EEG signals were spatially filtered using a large Laplacian filter centered on C3 in the extended 10-20 system.^48^^,^^49^ Every 100 ms, EEG signals of the latest 5 s were subjected to a third-order Butterworth bandpass (3-70 Hz) and notch (50 Hz) filter. After forward and backward filtering, the latest 1s data were subjected to Fourier transform with a Hanning window function to calculate signal strength in frequency space. This short-term Fourier transform analysis (STFT) resulted in a spectral band power. Using the signal power derived from the C3 electrode, SMR spectral power modulation was quantified as the size of ERSP using the following formula^21^:

$$ERSP\;\left(f,t\right)=100\times \frac{Power\;\left(f,t\right)-Reference\left(f\right)}{Reference\left(f\right)}$$

where f is frequency, t is time, Power is the signal strength, and Reference is the averaged power at the rest period. Then, SMR changes at the alpha-band (8-13 Hz) calculated by averaging the ERSP magnitude. The frequency bands used for online feedback were calibrated for each participant using frequency of interest (FOI) within the alpha-band. The FOI was determined from averaged ERSP during the open-loop block in Evaluation 1. Specifically, frequency that exhibited the most prominent negative at Task period in the alpha band was set as frequency of interest.^22^^,^^40^^,^^50^ The online calculated SMR magnitude at FOI was averaged for the latest 10 samples to determine instantaneous cortical activity without being affected by signal flicker.^22^^,^^40^^,^^43^

The online calculated SMR magnitude at FOI was used for the neurofeedback. The 25th to 75th percentiles of SMR in the open-loop block in Evaluation 1 were used to map the given SMR magnitude and visual feedback. The number of frame where stronger SMR magnitude (25th percentile) was associated with the abducted index finger (the 20th frame).

In the sham group, participants received yoked-sham neurofeedback where the provided feedback visual stimuli were associated with the SMR magnitude calculated for the previously measured EEG signal derived from the other participant.^44^ The identical procedure was conducted to determine the frame of the movie to present. Post-experiment debriefing did not reveal any participants who was aware of the intervention characteristic.

#### Offline EEG spectral power analysis

In the offline analysis on the whole-head scalp EEG data, we computed the cortical source activities using sLORETA algorithm^19^ implemented in Brainstorm toolbox^18^ using standard structural MRI and electrode positions. The estimated activities within M1 and S1 were subjected to the bandpass filtering and STFT analysis identical to the online processing.

To parameterize the power spectra, we used specparam algorithm^17^ and acquired IAF profiles and the aperiodic exponent which reflects the broadband 1/f component in the PSD. The ERSP values at IAF were computed for the offline analysis. In addition, whole-brain distributions of spectral power change were separately computed for each participant and averaged across the training blocks.

#### Offline SEP analysis

To calculate the amplitude of each SEP component, the acquired time series data were epoched into 600 ms data where each stimulus onset was centered. Then, the averaged data across epochs were z-scored using the average and standard deviation of amplitude before stimulus. Then, N9, N13 and N20 components were searched by finding the extrema within the range of ±3 ms of each latency.

#### Behavioral performance analysis

For each block, a successful trial was counted if the presented word was entered without error within the time limit, and the success rate for each movement time was calculated. Then, curve fitting using nonlinear least square algorithm was conducted for each participant success rate data using the following formula, y=aexp(−bx)+c where y and x represent the accuracy and condition, respectively. We fitted parameters a,b,andc and the parameter b was used to quantify the saturation speed.^51^

### Quantification and statistical analysis

All statistical results are reported in the results section of the main text and in the figure legends. Statistical tests were conducted using JASP.^52^ EEG-SMR features, SEP amplitude, and behavioral performance were analyzed using a mixed rmANOVA with Time and Group factors. The post-hoc analysis was conducted using *t*-test with Bonferroni correction. Before the data subjected to parametric tests, the normality and the equality of variance were tested by Shapiro-Wilk test and Levene test. Note that the sphericity of the variance was not confirmed because all factors in rmANOVA had two levels. As sensitivity analysis, the identical rmANOVA was applied to the intention-to-treat population and rmANOVA with aligned rank transform were performed.^27^ To ensure the absence of strong evidence in the alternative hypothesis, we additionally calculated Bayes factor of the null- and alternative hypothesis. Asterisks shown in figures indicates the statistical significance (corrected-*p* < 0.05).

### Additional resources

The protocol record was registered on ClinicalTrials.gov (NCT06814873: https://clinicaltrials.gov/study/NCT06814873).
