# Supplementary material for: Enhanced human sensorimotor integration via self-modulation of the somatosensory activity
Source: iScience. 2025 Mar 3;28(4):112145. doi: 10.1016/j.isci.2025.112145 (PMC11937678; doi:10.1016/j.isci.2025.112145)
Supplement: Document S1. Figures S1–S5 and Tables S1–S3 [file mmc1.pdf]

iScience, Volume *28*

Supplemental information

Enhanced human sensorimotor integration via self-modulation of the somatosensory activity

Seitaro Iwama, Takamasa Ueno, Tatsuro Fujimaki, and Junichi Ushiba

Supplementary Information

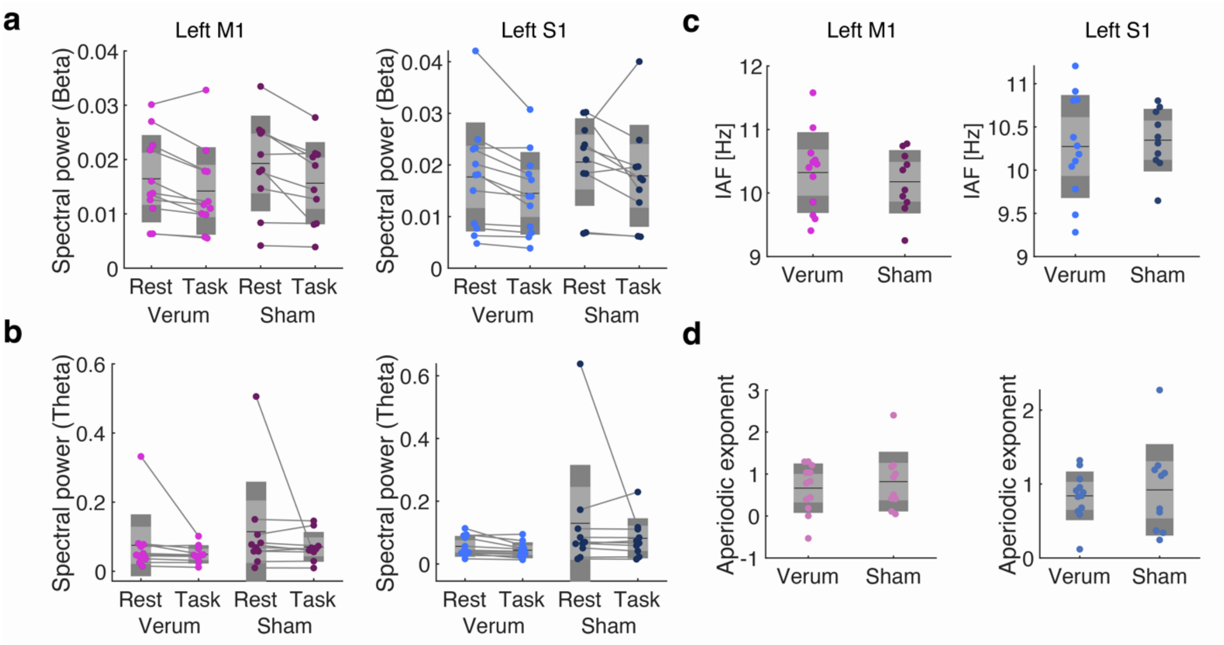

Supplementary Figure 1 Analysis for power spectral density features.

a. Spectral power at the beta-band at the rest and task periods in left M1 and S1. Each dot represents an individual participant. The mixed rmANOVA for M1 and S1 data indicated a significant main effect of condition (Rest and Task) (M1: $F = 22.0$, $p < 0.001$, $\eta^2 = 0.035$; S1: $F = 6.38$, $p = 0.02$, $\eta^2 = 0.026$). The post-hoc t-test revealed the significant decrease in the beta-band power at the task period (M1: $t = 4.69$, $p < 0.001$, $d = -0.37$; S1: t = 2.53, $p = 0.02$, $d = -0.32$). **b.** Spectral power at the theta-band at the rest and task periods in left M1 and S1. No significant main effects or interaction were found in both M1 and S1 data (M1: Condition, $F = 2.2$, $p = 0.15$, Group, $F = 1.1$, $p = 0.31$, Interaction: $F = 0.14$, $p = 0.71$; S1: Condition, $F = 1.55$, $p = 0.23$, Group, $F = 2.97$, $p = 0.1$, Interaction: $F = 0.53$, $p = 0.47$). **c.** Individual Alpha Frequency (IAF) comparison between Verum and Sham groups in left M1 and S1. No evidence in the systematic difference between groups were found in the left M1 or S1 (M1: $t = -0.21$, $p = 0.83$; S1: $t = -0.36$, $p = 0.72$). **d.** Aperiodic exponent comparison between Verum and Sham groups left M1 and S1. No evidence in the systematic difference between groups were found in the left M1 or S1 (M1: $t = -0.42$, $p = 0.70$; S1: $t = -0.52$, $p = 0.61$).

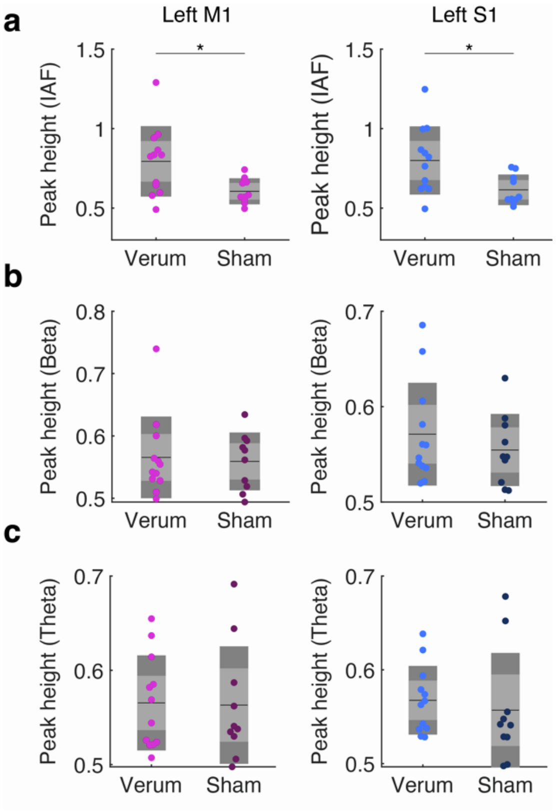

Supplementary Figure 2 Analysis for the parameterized spectral peak height.

a. Comparison of spectral peak heights at the individual alpha frequency (IAF) range for the Verum and Sham groups in the left M1 and S1. A significant difference in peak height is observed during the test period between the verum and sham groups in both regions (Two-sample *t*-test, Left M1: t = 2.79, p = 0.0014, d = 1.15; Left S1: t = 2.74, p = 0.0015, d = 1.14). The peak height was parameterized by the *specparam* algorithm. **b.** Comparison of spectral peak heights at the beta-band. (Two-sample *t*-test, Left M1: t = 0.09, p = 0.93; Left S1: t = 0.49, p = 0.64). **c.** Comparison of spectral peak heights at the theta-band. (Two-sample *t*-test, Left M1: t = 0.27, p = 0.79; Left S1: t = 86, p = 0.40).

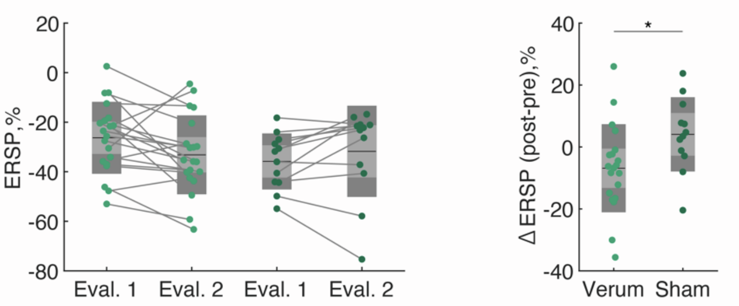

Supplementary Figure 3. Intention-to-treat population analysis for spectral power modulation

Comparison of event-related spectral perturbation (ERSP) during Evaluation 1 and Evaluation 2 for verum and sham groups in SM1, data from participants whose somatosensory evoked potential (SEP) were unavailable were included in the analysis. Each dot represents an individual participant, and lines connect the same participant's data across evaluations. The dark grey areas, light grey areas, and the black line represent 1 SD, 95% confidence interval, and mean values, respectively. The rmANOVA for intention-to-treat analysis indicated a significant interaction of time and group effects (F = 5.12, p = 0.031, η^2 = 0.032) and post-hoc two-sample t-test indicated the significant difference in the modulation size (p = 0.02, d = 0.84).

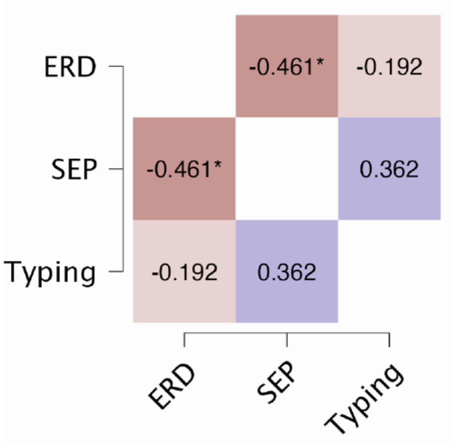

Supplementary Figure 4. Correlation heatmap for SMR-ERD, SEP and typing performance

An exploratory partial correlation analysis among the sensorimotor rhythm, SEP amplitude and behavioral performance modulated through the intervention. We found a significant positive correlation between ERD and SEP modulation (Pearson’s correlation: r = -0.461, p =0.031) while others did not reach statistical significance (ERD-Typing: r = -0.19, p = 0.39; SEP-Typing: r = 0.36, p = 0.098).

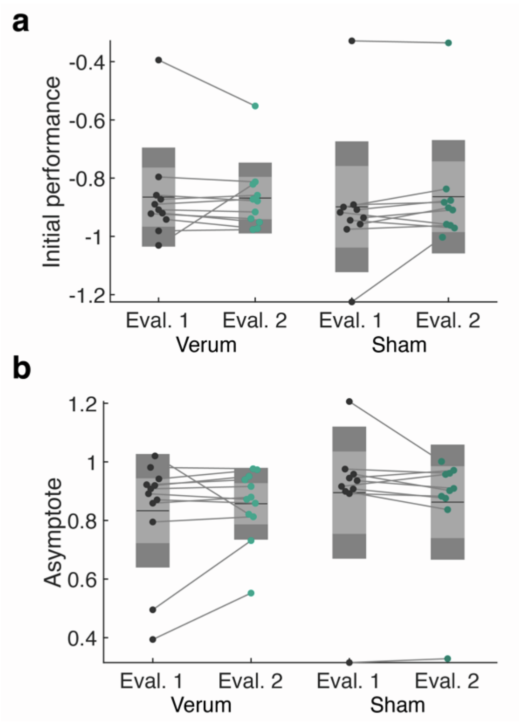

Supplementary Figure 5 Analysis for motor performance parameters.
a. Comparison of initial performance change for the verum and sham groups parameterized by the exponential fitting. The parameter *a* reflects the performance at the shorter movement time. No significant interaction and main effects were found by the rmANOVA analysis. **b.** Comparison of asymptote change for the verum and sham groups parameterized by the exponential fitting. The parameter *c* reflects the performance at the longer movement time. No significant interaction and main effects were found by the rmANOVA analysis.

Script for the instruction of kinesthetic motor imagery

First, perform abduction of the index finger. At this time, make sure that the muscles in your finger are engaged. Next, perform the same movement with about half the strength as before. Again, ensure that the muscles in your finger are engaged. Then, perform the same movement with about half the strength as in the previous step. Finally, without moving your finger, imagine the sensation you felt when performing the previous movements. You will repeat this mental imagery, referred to as kinesthetic motor imagery, during the task.Be careful not to actually engage the muscles in your finger during this process.

Statistical results for somatosensory evoked potentials

Supplementary Table 1. rmANOVA for N9 components

Within Subjects Effects

Cases	Sum of Squares	df	Mean Square	F	p	η²
Time	0.023	1	0.023	0.519	0.479	0.002
Time ✻ Group	0.097	1	0.097	2.184	0.155	0.008
Residuals	0.886	20	0.044			

Between Subjects Effects

Cases	Sum of Squares	df	Mean Square	F	p	η²
Group	1.433	1	1.433	3.07	0.095	0.122
Residuals	9.338	20	0.467			

Supplementary Table 2. rmANOVA for N13 components

Within Subjects Effects

Cases	Sum of Squares	df	Mean Square	F	p	η²
Time	0.208	1	0.208	1.617	0.218	0.013
Time ✻ Group	0.225	1	0.225	1.748	0.201	0.015
Residuals	2.576	20	0.129			

Between Subjects Effects

Cases	Sum of Squares	df	Mean Square	F	p	η²
Group	1.095	1	1.095	1.933	0.18	0.071
Residuals	11.336	20	0.567			

Supplementary Table 3. rmANOVA for N20 components

Within Subjects Effects

Cases	Sum of Squares	df	Mean Square	F	p	η^2
Time	0.097	1	0.097	2.691	0.117	0.018
Time ✻ Group	0.234	1	0.234	6.491	0.019	0.044
Residuals	0.722	20	0.036			

Between Subjects Effects

Cases	Sum of Squares	df	Mean Square	F	p	η^2
Group	0.047	1	0.047	0.22	0.644	0.009
Residuals	4.269	20	0.213			
